# Five new species of *Phintella* Strand, 1906 (Araneae, Salticidae) from the Wuling Mountains, China

**DOI:** 10.3897/zookeys.514.9159

**Published:** 2015-07-22

**Authors:** Yi Huang, Cheng Wang, Xian-Jin Peng

**Affiliations:** 1 College of Life Sciences, Hunan Normal University, Changsha, Hunan 410081, China; 2 Hunan Provincial Center for Disease Control and Prevention

**Keywords:** Jumping spider, southern Central China

## Abstract

Five new species of *Phintella* are described from the Wuling Mountains, China: *Phintella arcuata* **sp. n.** (male and female), *Phintella levii* **sp. n.** (female), *Phintella panda* **sp. n.** (female), *Phintella pulcherrima* **sp. n.** (male and female), and *Phintella wulingensis* **sp. n.** (female). Distribution data, detailed morphological characteristics, and illustrations of body and genital organs are presented.

## Introduction

*Phintella* was established by Strand in 1906 with the type species *Phintella bifurcilinea*. A total of 54 species has been reported mainly from the Oriental and Palaearctic regions (World Spider Catalog 2015), including 25 species transferred mainly from *Chrysilla* Thorell, 1887, *Telamonia* Thorell, 1887, *Icius* Simon, 1876, *Jotus* L. Koch, 1881 and 29 species described as new species. To date, there are 20 species known from China: *Phintella abnormis* (Bösenberg & Strand, 1906), *Phintella accentifera* (Simon, 1901), *Phintella aequipeiformis* (Zabka, 1985), *Phintella arenicolor* (Grube, 1861), *Phintella bifurcilinea* (Bösenberg & Strand, 1906), *Phintella cavaleriei* (Schenkel, 1963), *Phintella debilis* (Thorell, 1891), *Phintella hainani* Song et al. (1988), *Phintella linea* (Karsch, 1879), *Phintella longapophysis* (Lei & Peng, 2013), *Phintella longlinensis* (Lei & Peng, 2013), *Phintella parva* (Wesolowska, 1981), *Phintella popovi* (Proszyn’ski, 1979), *Phintella pygmaea* (Wesolowska, 1981), *Phintella suavis* (Simon, 1885), *Phintella suavisoides* (Lei & Peng, 2013), *Phintella tengchongensis* (Lei & Peng, 2013), *Phintella versicolor* (Koch, 1846), *Phintella vittata* (Koch, 1846) and *Phintella yinae* (Lei & Peng, 2013). The genus can be identified by: palpal tegulum with lobe and bump, embolus sets apically, usually short, pointed or furcated, tibia with one or more apophyses, female internal genitalia simple, coupulatory ducts of different length, usually not twisted, spermathecae round in most species (Zabka 2012).

The Wuling Mountains are located in southern Central China. All the areas are covered with folded mountains, the elevation generally above 1000 meters, the average temperature about 13.4 °C and the average precipitation reach to 1100–1600 millimeters. Vegetation are mainly composed of trees, forest coverage rate reached 80%. East-west of the mountains are range with karst geomorphology and stretch across Chongqing, Hunan, Hubei and Guizhou Provinces (Chen and Li 2003). Salticidae species richness in Wuling Mountains, up to now, more than 100 species including 6 known and several new species of *Phintella* have been collected. The present paper reports five new species of *Phintella* identified from the collections from Wuling Mountains.

## Material and methods

Descriptions were made based on specimens fixed in 75% ethanol. The specimens were examined and measured using an Olympus SZX16 stereomicroscope. The details were studied with an Olympus BX53 compound microscope. Male palp and female genitalia were drawn after they were dissected from the spiders. Photos were taken with a Canon PowerShot G12 digital camera mounted on an Olympus SZX16. Compound focus images were generated using Helicon Focus software.

All measurements are given in millimeters. Leg measurements are giving as total length (femur, patella + tibia, metatarsus, tarsus). Abbreviations used are as follows: AER anterior eye row; AERW anterior eye row width; ALE anterior lateral eyes; AME anterior median eyes; EL eye field length; PER posterior eye row, PERW posterior eye row; PLE posterior lateral eyes. Specimens are deposited in the College of Life Sciences, Hunan Normal University in Changsha, China.

## Taxonomy

### *Phintella* Strand, 1906

#### Phintella arcuata sp. n.

Taxon classificationAnimaliaAraneaeSalticidae

http://zoobank.org/0C74E8A6-EC9A-4601-BFFE-97833B79A042

Figs 1 , 2 , 3

##### Type material.

**Holotype**: ♂, **China, Hunan**: Shimen County, Huping mountain Township, Jinban Mountain Village, (29°26.288'N, 110°46.681'E, 554 m), 12 June 2014, C. Wang, B. Zhou, JH. Gan and YH. Gong leg. **Paratypes**: 1♀, Daling Village, (30°02'20.22N, 110°37'30.25E, 436 m), 18 October 2014, the collectors same as holotype; 1♀, Daling Village, (30°01'37.69N, 110°37'32.56E, 341 m), 19 October 2014, the collector same as holotype; 1♀, Daling Village, (30°01.681'N, 110°37.681'E, 677 m), 18 June 2014, the collectors same as holotype.

##### Etymology.

The specific name comes from the Latin *arcuata* (curved), referring to the form of yellow area at the middle part of male carapace.

##### Diagnosis.

**Figure 1.**
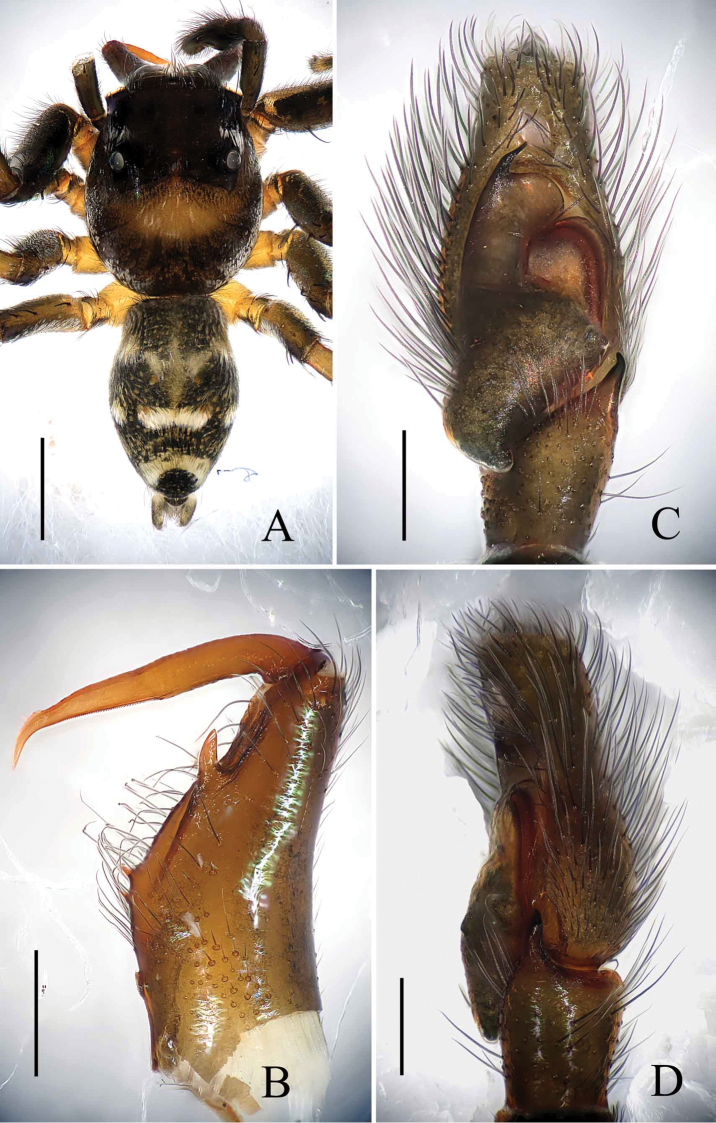
*Phintella arcuata* sp. n., **A** male body, dorsal view **B** left chelicerae of male, posterior view **C** male palp, ventral view **D** male palp, retrolateral view. Scale bars: 1.0 mm (**A**); 0.1 mm (**B**); 0.2 mm (**C–D**).

**Figure 2.**
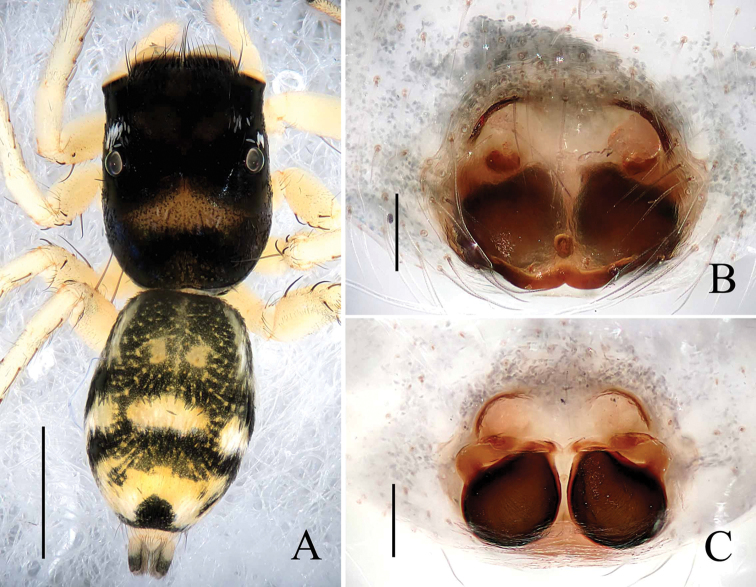
*Phintella arcuata* sp. n., **A** female body, dorsal view **B** epigyne, ventral view **C** vulva, dorsal view. Scale bars: 1.0 mm (**A**); 0.1 mm (**B–C**).

**Figure 3.**
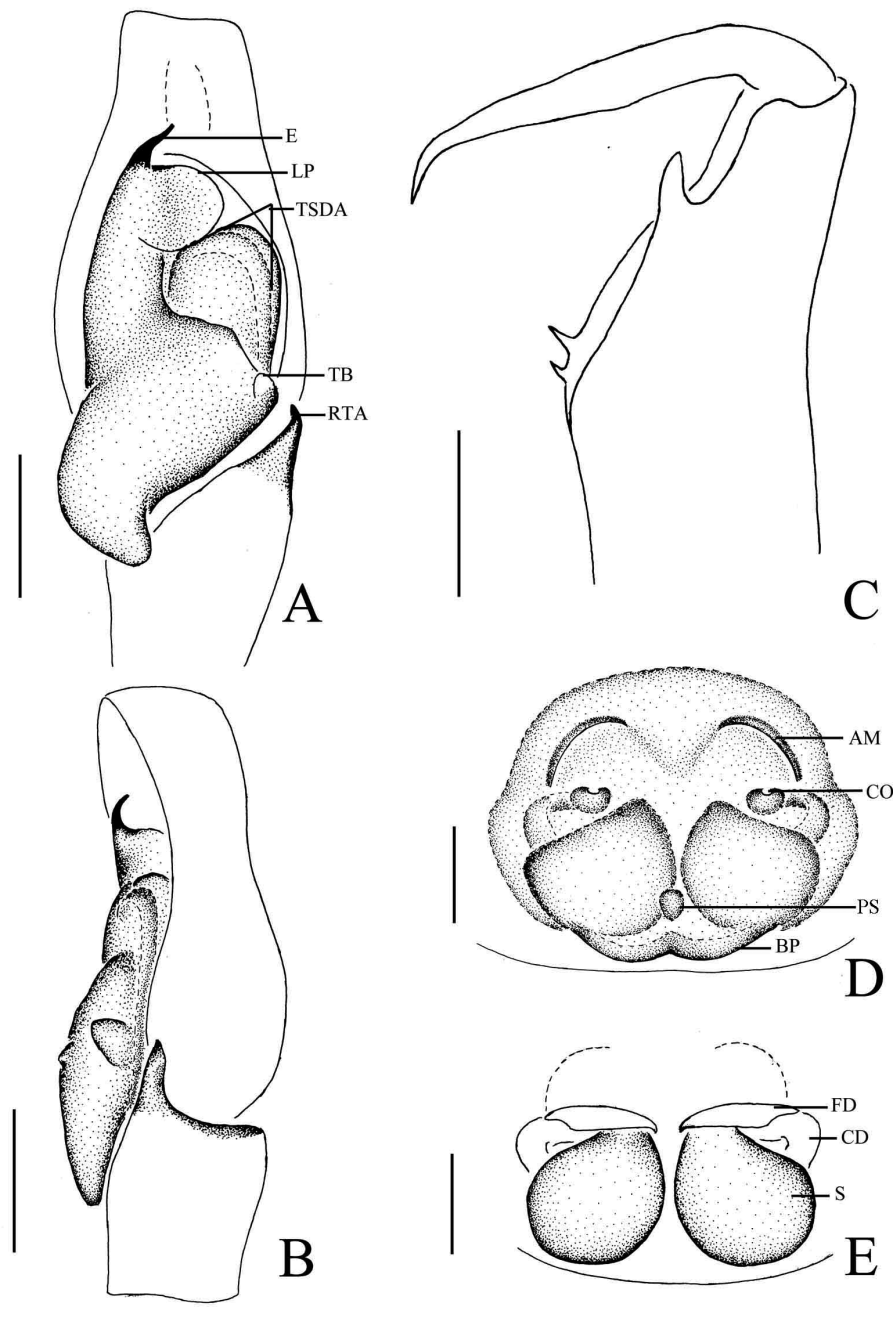
*Phintella arcuata* sp. n., **A** male palp, ventral view **B** male palp, retrolateral view **C** left chelicerae of male, posterior view **D** epigyne, ventral view **E** vulva, dorsal view. **AM** atrium margin **BP** basal plate **CD** copulatory duct **E** embolus **FD** fertilization duct **LP** lamellar process **PL** posterior lobe **PS** poriform structure **RTA** retrolateral tibial apophysis **TB** tegulum bump **TSDA** terminal sperm duct angle **S** spermathecae. Scale bars: 0.1 mm (**A–E**).

The male of this new species is very similar to *Phintella aequipeiformis* Zabka, 1985, especially in retrolateral view of male palp, but can be distinguished from the latter by: 1) the terminal sperm duct angle (TSDA) almost 60° (Fig. 3A) versus about 15° in *Phintella aequipeiformis*; 2) the distal end of retrolateral tibial apophysis curved in ventral view (Figs 1C, 3A) versus straight in *Phintella aequipeiformis*; 3) Lamellar process almost semicircular (Figs 1C, 3A) versus almost triangular in *Phintella aequipeiformis*; 4) dorsum of opisthosoma with 3 lines of markings, the first and second lines composed of 4 and 3 white stripes respectively (Fig. 1A) versus only with 2 lines in *Phintella aequipeiformis*. The female of this new species is similar to *Phintella linea* (Karsch, 1879), but can be distinguished from the latter by: 1) atrium margin distinct, located at the terminal portion of epigyne (Figs 2B, 3D) versus indistinct in *Phintella linea*; 2) spermathecae pyriform (Figs 2C, 3E) versus scutiform in *Phintella linea*; 3) spermathecae separated by less than one-seventh of their width in dorsal view (Figs 2C, 3E) versus about one-third of their width in *Phintella linea*; 4) base of fertilization ducts extend beyond the base of copulatory ducts in dorsal view (Figs 2C, 3E) versus almost at same level in *Phintella linea*.

##### Description.

**Male**: Total length 4.20. Prosoma 2.15 long, 1.75 wide. Opisthosoma 2.05 long, 1.30 wide. Clypeus 0.14 high. Carapace (Fig. 1A) blackish-brown, inflated, covered with white and brown long hair. Bilateral of eye field and posterior sides of carapace with white curved stripes covered by white hair, anterior of thorax with a curved yellowish area behind eye field. Eye bases and margins of carapace black. Fovea reddish-brown, longitudinal, cervical and radial grooves indistinct. Eye sizes and inter-distances: AME 0.50, ALE 0.31, PLE 0.28, AERW 1.35, PERW 1.40, EL 1.03. Chelicerae (Figs 1B, 3C) dark brown, with 2 promarginal teeth and 1 retromarginal. Endites broader at base, anterior margin with bristles. Labium dark brown, with brown thin hair. Sternum colored as labium, anteriorly straight and posteriorly subacute, with thin hair. Leg trochanters, coxae and tarsi yellowish-brown, others dark brown. Leg spinnation: tibiae I and II with three pairs, metatarsi I and II with two pairs of long spines. Measurements of legs: I 7.16 (2.05, 3.01, 1.55, 0.55), II 5.55 (1.70, 2.10, 1.20, 0.55), III 5.70 (1.75, 1.95, 1.45, 0.55), IV 5.80 (1.80, 2.00, 1.45, 0.55). Leg formula: 1432. Dorsum of opisthosoma (Fig. 1A) long oval, anterior area with two pairs of white stripes, median area with two pairs of muscle impressions and three transverse white stripes, posterior end with one cambered white stripes, covered with light dots. Venter pale brown, with four longitudinal lines formed by light dots at middle part.

Palp (Figs 1C–D, 3A–B): tibia slightly longer than wide, retrolateral apophysis thin, with a swollen base and slightly curved tip. Posterior lobe large, curved at terminal end and slightly sharp at the tip. Tegulum bump situated posteriorly, almost triangular in retrolateral view. Embolus thin, originated at the top on tegulum, the tip almost extended to the position of 1:00 o’clock. Lamellar process big, almost semicircular. Sperm duct visible and the terminal sperm duct angle almost 60°.

**Female**: Total length 4.10. Prosoma1.97 long, 1.41 wide. Opisthosoma 2.06 long, 1.43 wide. Clypeus 0.14 high. Carapace (Fig. 2A) dark brown, anterior margin covered with dark brown hair. Bilateral of eye field with white stripes formed by white hair, anterior median of thorax with a triangular yellowish area behind eye field. Margins of carapace and eye bases black. Eye sizes and inter-distances: AME 0.48, ALE 0.27, PLE 0.26, AERW 1.41, PERW 1.33, EL 1.02. Fovea reddish-brown, longitudinal, cervical and radial grooves indistinct. Chelicerae, endites, labium, sternum similar to male except for the lighter color. Legs yellow. Leg spinnation: as same as male. Measurements of legs: I 3.05 (0.95, 1.20, 0.50, 0.40), II 2.90 (0.90, 1.10, 0.50, 0.40), III 3.45 (1.00, 1.30, 0.75, 0.40), V 4.00 (1.25, 1.45, 0.90, 0.40). Leg formula: 4312. Dorsum of opisthosoma (Fig. 2A) pale brown, the markings similar to male, covered with light dots. Venter grey brown, bilateral of posterior portion with two longitudinal white stripes, and two longitudinal lines formed by light dots behind epigastric furrow.

Epigyne (Figs 2B–C, 3D–E) with arc band-shaped atrium margins anteriorly. Copulatory openings small, situated at the median area, the distance between them about equal to spermathecal width. Basal plate arched, with wave-like protruding parts. Copulatory ducts slightly thick, curved at middle part. Spermathecae pyriform, close to each other, separated by less than one-tenth of their width.

##### Distribution.

China (Hunan).

#### Phintella levii sp. n.

Taxon classificationAnimaliaAraneaeSalticidae

http://zoobank.org/5C5C50FD-C08D-40FA-884C-ED5129EC25CB

Figs 4 , 5

##### Type material.

**Holotype**: ♀, **China, Hunan**: Shimen County, Hupingshan Township, Quanping Village, (30°00.786'N, 110°35.822'E, 611 m), 15 June 2014, C. Wang, B. Zhou, JH. Gan and YH. Gong leg. **Paratypes**: 1♀, same data as Holotype.

##### Etymology.

The specific name is in honor of Dr. H. Levi. a famous American arachnoid scholar.

##### Diagnosis.

**Figure 4.**
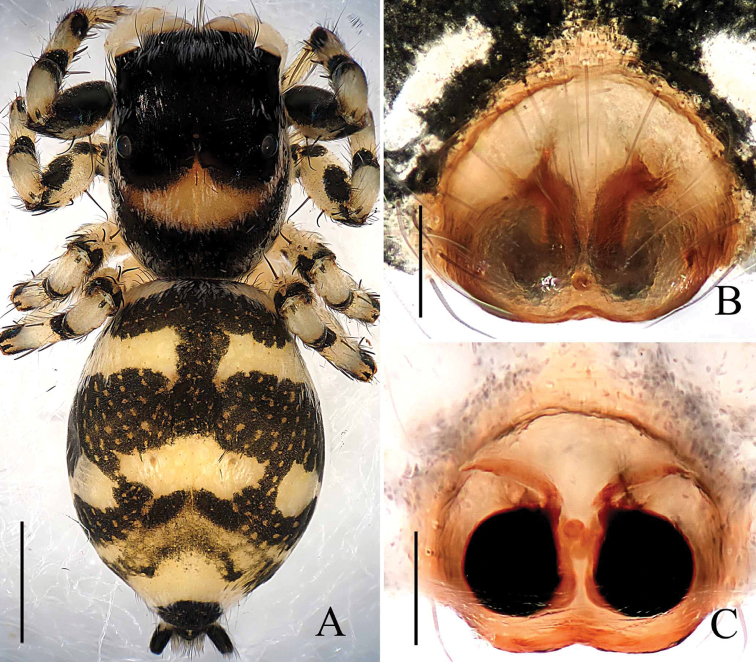
*Phintella levii* sp. n., **A** female body, dorsal view **B** epigyne, ventral view **C** vulva, dorsal view. Scale bars: 1.0 mm (**A**); 0.1 mm (**B–C**).

**Figure 5.**
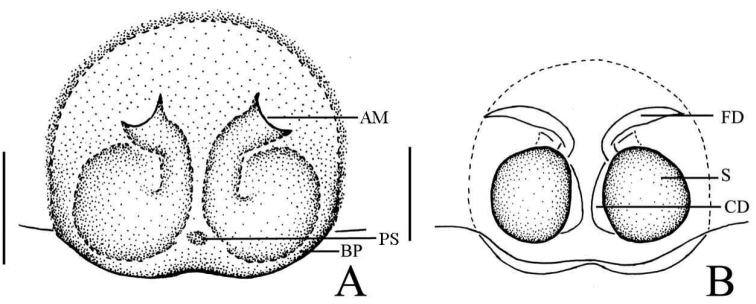
*Phintella levii* sp. n., **A** epigynum, ventral view **B** vulva, dorsal view. **AM** atrium margin **BP** basal plate **CD** copulatory duct **FD** fertilization duct **PS** poriform structure **S** spermathecae. Scale bars: 0.1 mm (**A–B**).

This new species is similar to *Phintella nigirica* Proszyn’ski, 1992 in having copulatory ducts originated from spermathecal base and the terminal part inflated, but can be distinguished from the latter by: 1) epigyne almost round (Figs 4B, 5A) versus triangular in *Phintella nigirica*; 2) the distance between atrium margins wider than half of spermathecal width in ventral view (Figs 4B, 5A) versus distinctly narrower than half of spermathecal width in *Phintella nigirica*; 3) copulatory ducts about half length of epigyne in ventral view (Figs 4B, 5A) versus distinctly longer than half length of epigyne in *Phintella nigirica*; 4) epigyne with poriform structure situated at the area between bases of two spermathecae (Figs 4B, 5A) versus absent in *Phintella nigirica*; 5) spermathecae pyriform (Figs 4C, 5B) versus scutiform in *Phintella nigirica*; 6) dorsum of opisthosoma with yellow stripes and brown stripes alternately arranged (Fig. 4A) versus with two dark submarginal streaks with brown scales along edges in *Phintella nigirica*.

##### Description.

**Female**: Total length 4.04. Prosoma 1.61 long, 1.11 wide. Opisthosoma 2.35 long, 1.71 wide. Clypeus 0.15 high. Carapace (Fig. 4A) blackish-brown, covered with long brown and white hair. Bilateral of eye field and posterior sides of carapace with white curved stripes covered by white hair, middle part of carapace with one a W-shaped yellowish brown area. Margin of carapace and eye bases black. Fovea reddish-brown, longitudinal, cervical and radial grooves indistinct. Eye sizes and inter-distances: AME 0.46, ALE 0.22, PLE 0.24, AERW 1.11, PERW 1.05, EL 0.88. Chelicerae yellowish-brown, with 2 promarginal teeth and 1 retromarginal. Endites dark brown, with narrower base, anterior margin with bristles. Labium colored as endites, broader at base, covered with black hair at terminal. Sternum dark brown, anteriorly straight and posteriorly subacute, covered with thin hair. Terminal part of femur, anterior and terminal parts of patella with dark annuli, others yellow. Leg spinnation: tibiae I and II with two pairs, metatarsi I and II also with two pairs of long spines. Measurements of legs: I 3.00 (0.95, 1.10, 0.55, 0.40), II 2.80 (0.90, 1.00, 0.50, 0.40), III 3.45 (1.10, 1.15, 0.80, 0.40), IV 3.90 (1.20, 1.40, 0.90, 0.40). Leg formula: 4312. Dorsum of opisthosoma (Fig. 4A) oval, yellow stripes and brown stripes alternately arranged, median area with two pairs of muscle impressions. Venter brown, the middle part with one broad longitudinal pale brown stripe covered with two longitudinal lines formed by light dots. Spinnerets black.

Epigyne (Figs 4B–C, 5A–B) almost round. Atrium margins situated at anterior-median area. Basal plate big, below epigastric furrow. Copulatory ducts long, about half length of epigyne, and the terminal part slightly inflated. Spermathecae pyriform, separated by one-third of their width.

**Male**: unknown.

##### Distribution.

China (Hunan).

#### Phintella panda sp. n.

Taxon classificationAnimaliaAraneaeSalticidae

http://zoobank.org/02F5BDC9-EE3A-453F-92CF-5E813C96872D

Figs 6 , 7

##### Type material.

**Holotype**: ♀, **China, Hunan**: Shimen County, Hupingshan Township, Daling Village, (30°02.359'N, 110°37.301'E, 892 m), 19 June 2014, C. Wang, B. Zhou, JH. Gan and YH. Gong leg.

##### Etymology.

The specific name comes from the Latin *panda* (panda), referring to the form of markings between the posterior lateral eyes, which is similar to the markings of the pandas’ eyes..

##### Diagnosis.

**Figure 6.**
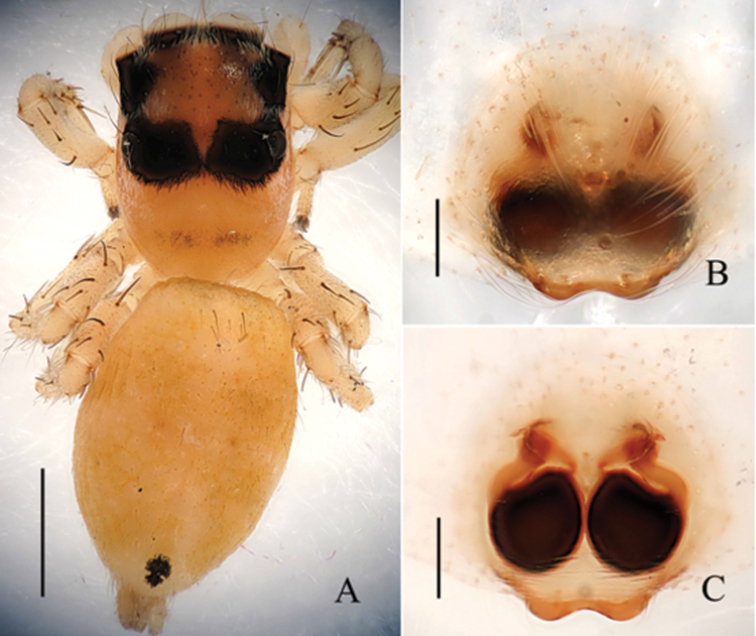
*Phintella panda* sp. n., **A** female body, dorsal view **B** epigyne, ventral view **C** vulva, dorsal view. Scale bars: 1.0 mm (**A**); 0.1 mm (**B–C**).

**Figure 7.**
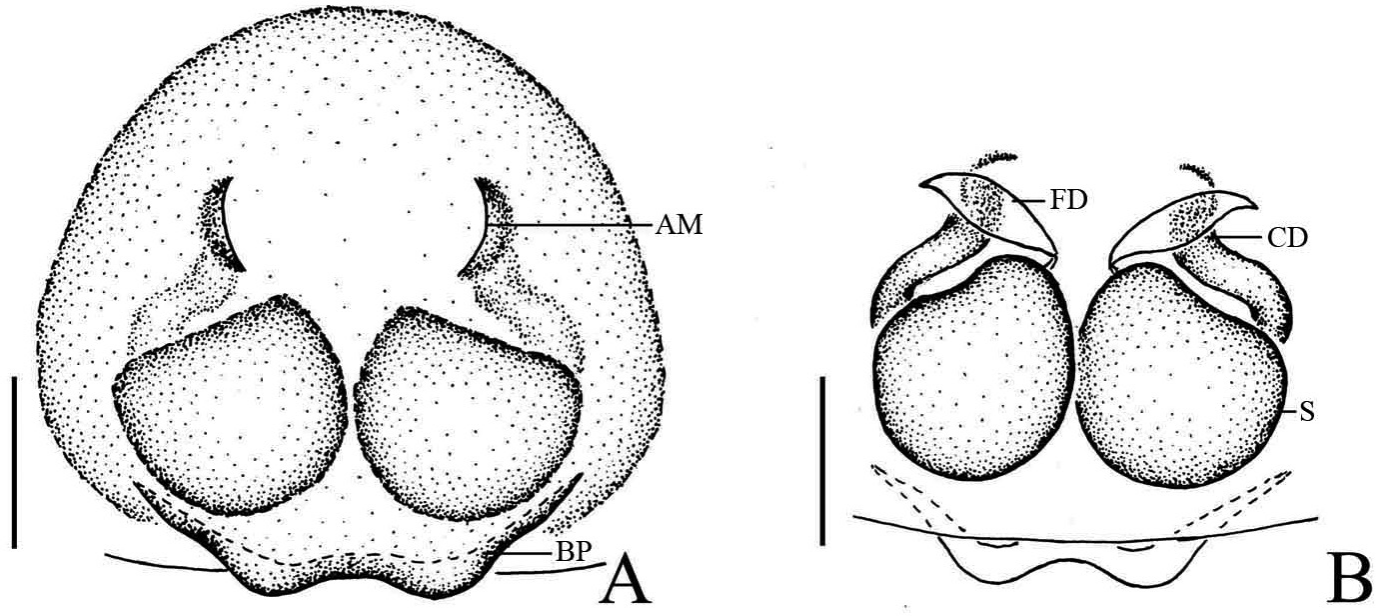
*Phintella panda* sp. n., **A** epigynum, ventral view **B** vulva, dorsal view. **AM** atrium margin **BP** basal plate **CD** copulatory ducts **FD** fertilization duct **S** spermathecae. Scale bars: 0.1 mm (**A–B**).

This new species is somewhat similar to *Phintella arcuata* sp. n. in having pyriform spermathecae and a similar basal plate, but can be distinguished from the latter by: 1) atrium margins slit-like, longitudinal (Figs 6B, 7A) versus arc band-shaped, diagonal in *Phintella arcuata*; 2) base of spermathecae far from basal plate in dorsal view (Figs 6C, 7B) versus almost at same level in *Phintella arcuata*; 3) distance between the two protruding parts of basal plate wider distinctly, and the protruding parts hornlike (Figs 6B, 7A) versus wave-like in *Phintella arcuata*; 4) spermathecae touching each other in middle part (Figs 6C, 7B) versus separated distinctly in *Phintella arcuata*; 5) dorsum of opisthosoma with only one black spot (Fig. 6A) versus with complicated markings in *Phintella arcuata*.

##### Description.

**Female**: Total length 4.68. Prosoma 1.96 long, 1.36 wide. Opisthosoma 2.54 long, 1.61 wide. Clypeus 0.15 high. Carapace (Fig. 6A) yellowish-brown, color of cephalic region darker, with one pair of black markings between PER bases. Eye bases black, eye field covered with sparse yellowish-brown bristles, denser in vicinity of eyes. Fovea short and thin, reddish-brown, longitudinal, cervical and radial grooves indistinct. Eye sizes and inter-distances: AME 0.45, ALE 0.23, PLE 0.25. AERW 1.38, PERW 1.30, EL 1.14. Chelicerae yellow, with 2 promarginal teeth and 1 retromarginal. Endites narrower at base, anterior margin with bristles. Labium broader at base, covered with brown thin hair, denser in anterior part. Sternum pale yellow, anteriorly straight and posteriorly subacute, covered with brown thin hair. Legs pale yellow to yellow. Leg spinnation: tibiae I and II with three pairs, metatarsi I and II with two pairs of long spines. Measurements of legs: I 3.01 (0.93, 1.15, 0.50, 0.43), II 2.88 (0.90, 1.05, 0.50, 0.43), III 3.58 (1.05, 1.20, 0.90, 0.43), IV 3.78 (1.15, 1.30, 0.90, 0.43). Leg formula: 4312. Dorsum of opisthosoma (Fig. 6A) long oval, yellow, with lighter area, covered with sparse thin hair, median area with two pairs of muscle impressions, posterior area with one black spot. Venter pale yellow, without distinct marking.

Epigyne (Figs 6B–C, 7A–B) slightly longer than wide. Atrium margins slit-like, longitudinal, situated anteriorly. Basal plate arched, with two protruding parts below epigastric furrow. Copulatory ducts long and thick, originated from the middle part of outer margin of spermathecae, slightly snaky. Spermathecae pyriform, touching each other in the middle section.

**Male**: unknown.

##### Distribution.

China (Hunan).

#### Phintella pulcherrima sp. n.

Taxon classificationAnimaliaAraneaeSalticidae

http://zoobank.org/B66C79A1-15E6-4F25-86F4-6D936B105624

Figs 8 , 9 , 10

##### Type material.

**Holotype**: ♂, **China, Guizhou**: Tongren City, Wenbi Mountains, (27°43.168'N, 109°10.077'E, 475 m), 26 July 2014, XQ. Mi, Y. Huang, C. Wang, B. Zhou and MY. Liao leg. **Paratypes**: 3♀7♂, same data as Holotype.

##### Etymology.

The specific name comes from the Latin *pulcherrima* (very beautiful), referring to the beautiful appearance of the specimens of this new species in alcohol.

##### Diagnosis.

**Figure 8.**
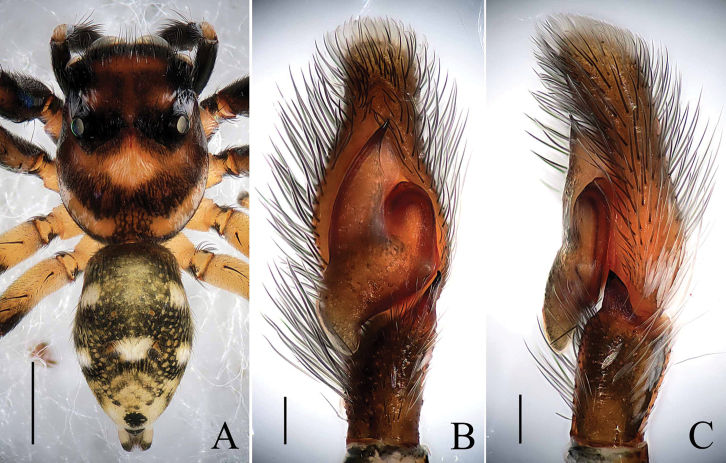
*Phintella pulcherrima* sp. n., **A** male body, dorsal view **B** male palp, ventral view **C** male palp, retrolateral view. Scale bars: 1.0 mm (**A**); 0.1 mm (**B–C**).

**Figure 9.**
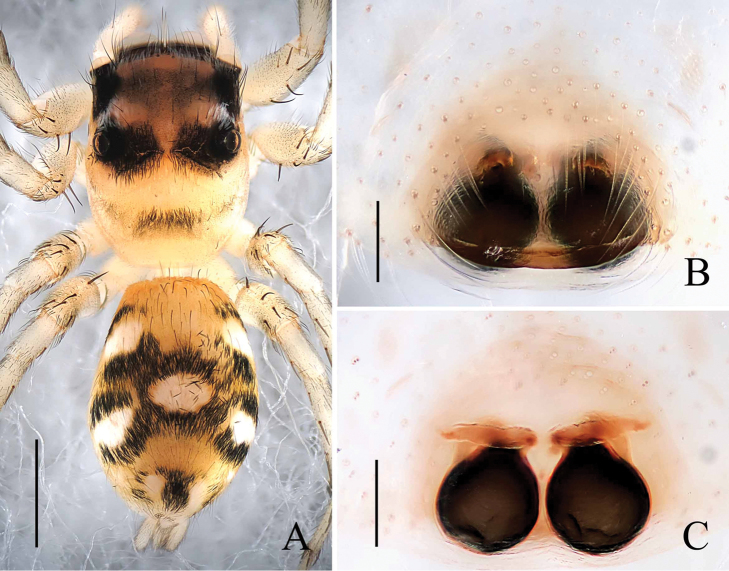
*Phintella pulcherrima* sp. n., **A** female body, dorsal view **B** epigyne, ventral view **C** vulva, dorsal view. Scale bars: 1.0 mm (**A**); 0.1 mm (**B–C**).

**Figure 10.**
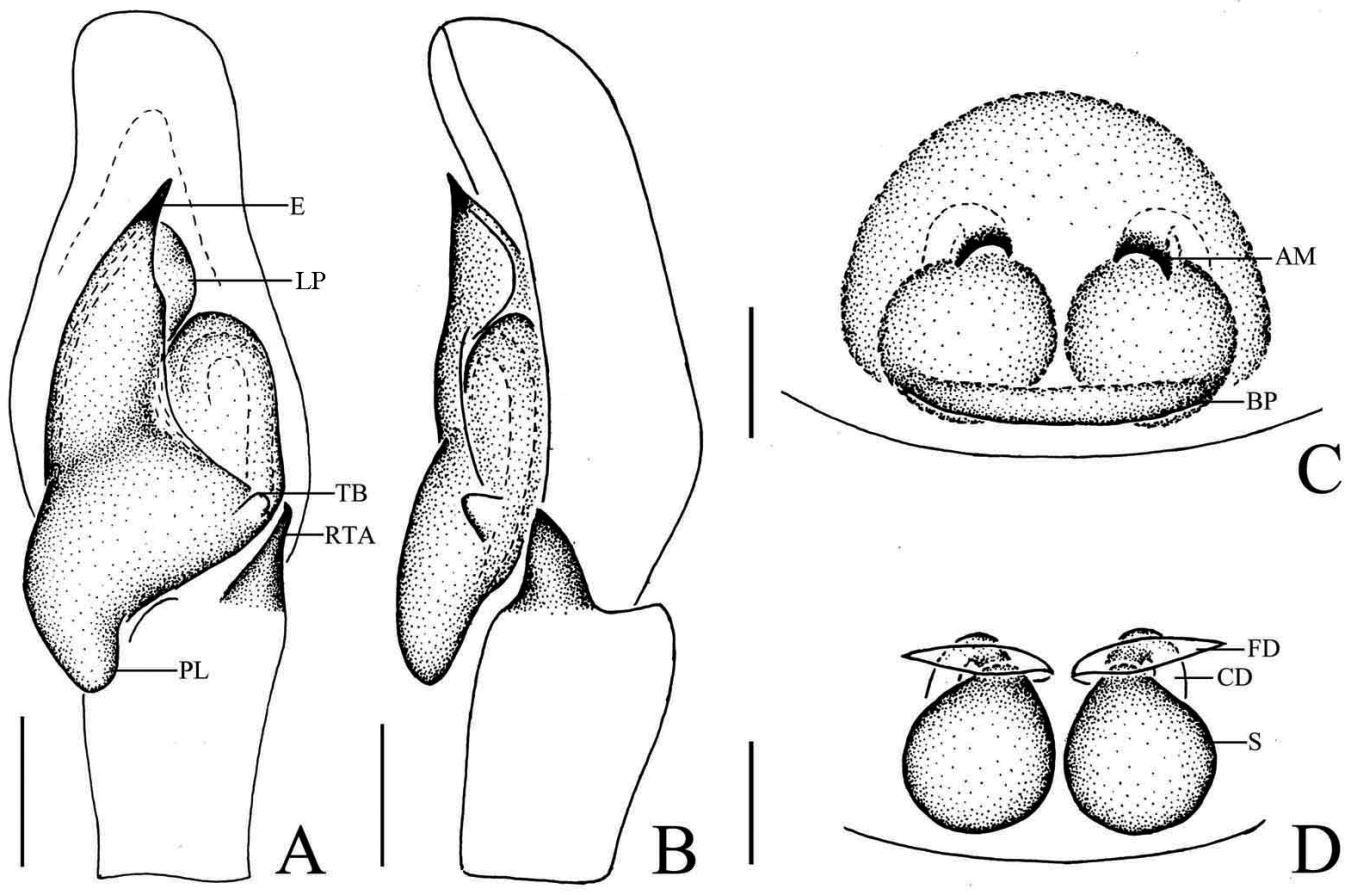
*Phintella pulcherrima* sp. n., **A** male palp, ventral view **B** male palp, retrolateral view **C** epigyne, ventral view **D** vulva, dorsal view. Scale bars: 0.1 mm (**A**–**D**). **AM** atrium margin **BP** basal plate **CD** copulatory ducts **E** embolus **FD** fertilization ducts **LP** lamellar process **PL** posterior lobe **RTA** retrolateral tibial apophysis **TB** tegulum bump; **S** spermathecae.

This new species is very similar to *Phintella linea* (Karsch, 1879) in having similar palps and epigynes, but the males can be distinguished from the latter by: 1) tibia slender relatively, longer than wide (Figs 8B, 10A) versus dumpy, wider than long in *Phintella linea*; 2) the posterior lobe only extending to tibial terminal in ventral view (Figs 8B, 10A) versus extending to tibial base in *Phintella linea*; 3) the distal end of retrolateral tibial apophysis curved in ventral view (Figs 8B, 10A) versus straight in *Phintella linea*; 4) dorsum of opisthosoma with several white round markings and covered with light dots (Fig. 8A) versus with dark brown pattern composed of longitudinal and diagonal stripes in *Phintella linea*. The females can be distinguished from the latter by: 1) spermathecae almost spherical (Figs 9C, 10D) versus pyriform in *Phintella linea*; 2) the distance between copulatory openings narrower than spermathecal width in ventral view (Figs 9B, 10C) versus almost equal to spermathecal width in *Phintella linea*; 3) epigyne with a broad, band-shaped basal plate (Figs 9B, 10C) versus the basal plate divided into three parts in *Phintella linea*; 4) markings on dorsum of opisthosoma (Fig. 9A) also different.

##### Description.

**Male**: Total length 4.63. Prosoma 2.37 long, 1.78 wide. Opisthosoma 2.26 long, 1.42 wide. Clypeus 0.15 high. Carapace (Fig. 8A) reddish-brown, widest at coxae II and III. Posterior margins of carapace with yellow curved stripes covered with whiter hair, anterior median of thorax with a quadrangular yellowish area covered by white hair. Eye field with black patches medially, white hair posterior-bilaterally situated, covered with sparse brown hair, denser in eye bases. Fovea reddish-brown, longitudinal, cervical and radial grooves indistinct. Eye sizes and inter-distances: AME 0.51, ALE 0.29, PLE 0.29, AERW 1.55, PERW 1.43, EL 1.21. Chelicerae reddish-brown, with 2 promarginal teeth and 1 retromarginal. Endites yellowish-brown, with broader bases, anterior margin with bristles. Labium dark brown, covered with brown thin hair, denser in anterior part. Sternum yellow, anteriorly straight and posteriorly curved. Legs I and II dark brown except middle of patella, metatarsi and tarsus yellow; Legs III and IV yellow except terminal of femur, middle of tibia and terminal of metatarsi dark brown. Leg spinnation: tibiae I and II with three pairs, metatarsi I and II with two pairs of long spines. Measurements of legs: I 7.35 (2.15, 3.05, 1.55, 0.60), II 5.85 (1.80, 2.25, 1.25, 0.55), III 6.05 (1.85, 2.10, 1.55, 0.55), IV 6.45 (1.95, 2.15, 1.75, 0.60). Leg formula: 1432. Dorsum of opisthosoma (Fig. 8A) long oval, anterior-bilateral area with one pair of round white markings, median area with two pairs of muscle impressions and three white markings, posterior end with two white markings separated by a black spot, covered with light dots. Venter pale brown, with four longitudinal lines formed by light dots at middle part.

Palp (Figs 8B–C, 10A–B): tibia longer than wide distinctly, retrolateral apophysis thin, with a swollen base and slightly curved tip in ventral view, broad base and sharp tip in retrolateral view. Poster lobe big, terminal curved and the tip blunt. Tegulum bump situated posteriorly, almost at same level with the tip of retrolateral tibial apophysis in ventral view, almost triangular in retrolateral view. Embolus thin, short, originated from top of bulb, the tip about pointed to the position of 1:00 o’clock. Lamellar process small relatively, almost crescent. Sperm ducts visible, running submarginally along retrolateral margin of tegulum in ventral view.

**Female**: Total length 4.45. Prosoma 2.04 long, 1.48 wide. Opisthosoma 2.31 long, 1.59 wide. Clypeus 0.15 high. Carapace (Fig. 9A) yellowish-brown, darker in cephalic region. Sparse brown bristles on eye field, denser in vicinity of eyes. Posterior bilateral of eye field with white hair and big brown spots between PLE bases. Fovea, cervical and radial grooves indistinct. Eye sizes and inter-distances: AME 0.49, ALE 0.28, PLE 0.28, AERW 1.45, PERW 1.36, EL 1.09. Chelicerae, endites, labium, sternum similar to male except the lighter color. Legs yellow. Leg spinnation: as same as male. Measurements of legs: I 4.15 (1.30, 1.70, 0.70, 0.45), II 3.90 (1.25, 1.50, 0.70, 0.45), III 4.45 (1.45, 1.55, 1.00, 0.45), IV 5.00 (1.55, 1.75, 1.25, 0.45). Leg formula: 4312. Dorsum of opisthosoma (Fig. 9A) yellow, the markings similar to male except the white markings around with black area. Venter pale yellow.

Epigyne (Figs 9B–C, 10C–D) slightly wider than long. Atrium margins curved, the distance between them less than spermathecal width. Basal plate band-shaped, slightly curved. Copulatory ducts short, curved at middle part and most parts covered by spermathecae and fertilization ducts. Spermathecae almost spherical, close to each other, separated by less than one-twelfth of their width.

##### Distribution.

China (Guizhou).

#### Phintella wulingensis sp. n.

Taxon classificationAnimaliaAraneaeSalticidae

http://zoobank.org/D0D2987D-2D40-4BD0-9601-88E953C9C338

Figs 11 , 12

##### Type material.

**Holotype**: ♀, **China, Guizhou**: Songtao County, Fanjing Mountains national native reserve, Wuluo Township, Taoyuan Village, (28°00'0113N, 108°46'4784E, 880 m), 31 July 2014, XJ. Peng, Y. Huang, P. Liu, C. Wang, B. Zhou and MY. Liao leg. **Paratypes**: 1♀, **Hunan**: Shimen County, Hupingshan Township, Daling Village, (30°01.681'N, 110°37.681'E, 677 m), 18 June 2014, C. Wang, B. Zhou, JH. Gan and YH. Gong leg; 1♀, Daling Village, (30°02.175'N, 110°37.455'E, 710 m), 19 June 2014, C. Wang, B. Zhou, JH. Gan and YH. Gong leg.

##### Etymology.

The specific name refers to the type locality; the Wuling Mountains.

##### Diagnosis.

**Figure 11.**
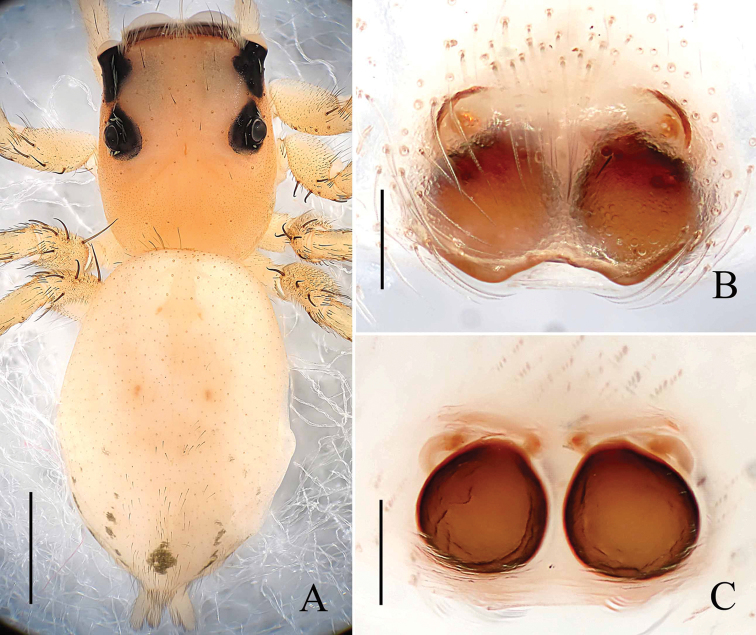
*Phintella wulingensis* sp. n., **A** female body, dorsal view **B** epigyne, ventral view **C** vulva, dorsal view. Scale bars: 1.0 mm (**A**); 0.1 mm (**B–C**).

**Figure 12.**
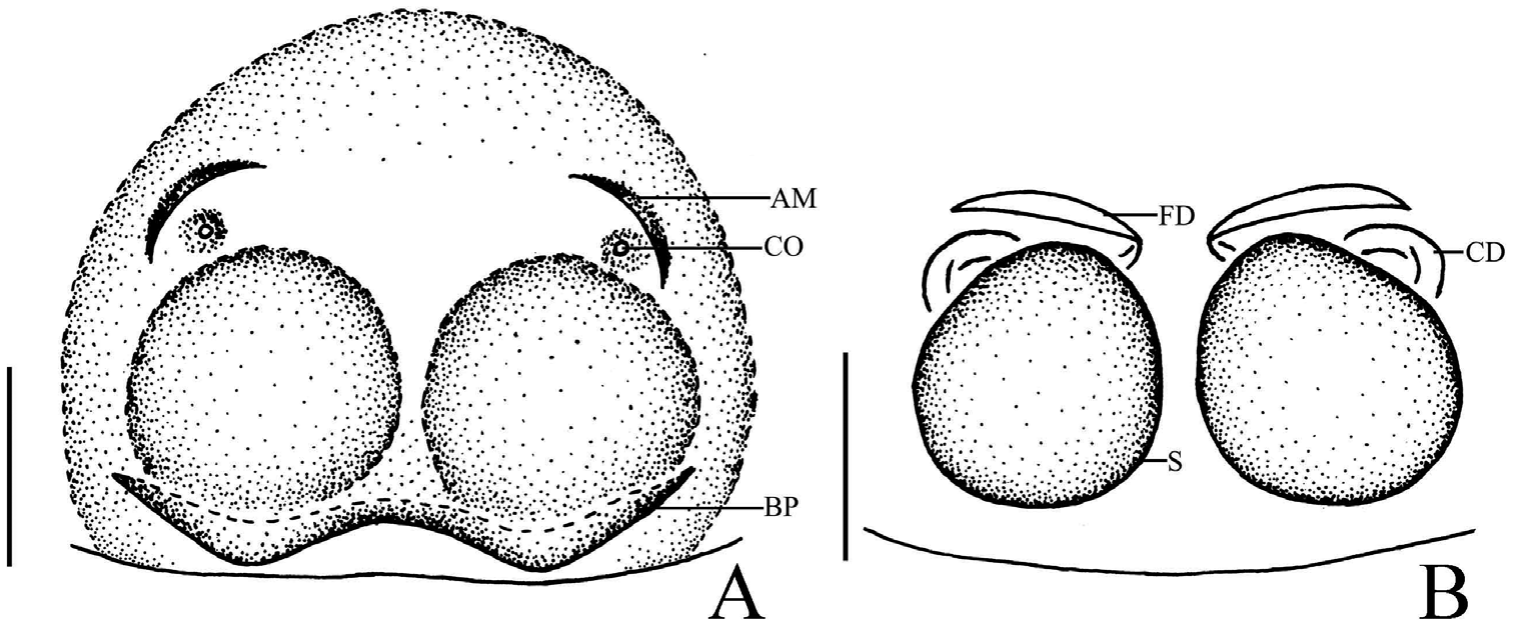
*Phintella wulingensis* sp. n., **A** epigynum, ventral view **B** vulva, dorsal view. **AM** atrium margin **BP** basal plate **CD** copulatory duct **CO** copulatory opening **FD** fertilization duct **S** spermathecae. Scale bars: 0.1 mm (**A–B**).

This new species is somewhat similar to *Phintella panda* sp. n. in having a similar appearance and epigyne with an arched basal plate, but can be distinguished from the latter by: 1) atrium margins diagonal (Figs 11B, 12A) versus longitudinal in *Phintella panda*; 2) copulatory openings round (Figs 11B, 12A) versus invisible in *Phintella panda*; 3) situation of atrium margins close to the top of spermathecae (Figs 11B, 12A) versus far from the top of spermathecae in *Phintella panda*; 4) copulatory ducts thinner, narrower than one-tenth of spermathecal width (Figs 11C, 12B) versus about two-seventh of spermathecal width in *Phintella panda*; 5) spermathecae almost spherical (Figs 11C, 12B) versus pyriform in *Phintella panda*; 6) carapace without markings (Fig. 11A) versus with one pair of black markings between PER bases in *Phintella panda*.

##### Description.

**Female**: Total length 5.07. Prosoma 1.96 long, 1.55 wide. Opisthosoma 3.07 long, 2.07 wide. Clypeus 0.16 high. Carapace (Fig. 11A) yellow, cephalic region square and thoracic region acutely declining. Eye bases black except PME bases brown, eye field covered with sparse brown hairs. Fovea thin and short, longitudinal, cervical and radial grooves indistinct. Eye sizes and inter-distances: AME 0.52, ALE 0.29, PLE 0.28, AERW 1.52, PERW 1.41, EL 1.04. Chelicerae yellow, with 2 promarginal teeth and 1 retromarginal. Endites narrower at base, anterior margin with bristles, almost parallel. Labium yellow, hair dark and thin, denser in anterior area. Sternum anteriorly straight and posteriorly subacute, covered with brown thin hair. Legs pale yellow to yellow. Leg spinnation: tibiae I and II with three pairs, metatarsi I and II with two pairs of long spines. Measurements of legs: I 3.38 (1.05, 1.30, 0.60, 0.43), II 3.23 (1.00, 1.25, 0.55, 0.43), III 3.73 (1.15, 1.35, 0.80, 0.43), IV 4.23 (1. 45, 1.45, 0.90, 0.43). Leg formula: 4312. Dorsum of opisthosoma (Fig. 11A) long oval, pale yellow, median area with two pairs of muscle impressions, posterior area with small brown spots, covered with recumbent hair. Venter pale yellow, without distinct markings.

Epigyne (Figs 11B–C, 12A–B) slightly wider than long, atrium margins curved, diagonal, situated anteriorly. Copulatory openings small, situated anteriorly, separated from each other distinctly. Basal plate arched, with two protruding parts close to epigastric furrow. Copulatory ducts thin, narrower than one-tenth of spermathecal width, curved at middle part. Spermathecae almost spherical, close to each other, separated by less than two-seventh of their width.

**Male**: unknown.

##### Distribution.

China (Guizhou, Hunan).

**Figure 13.**
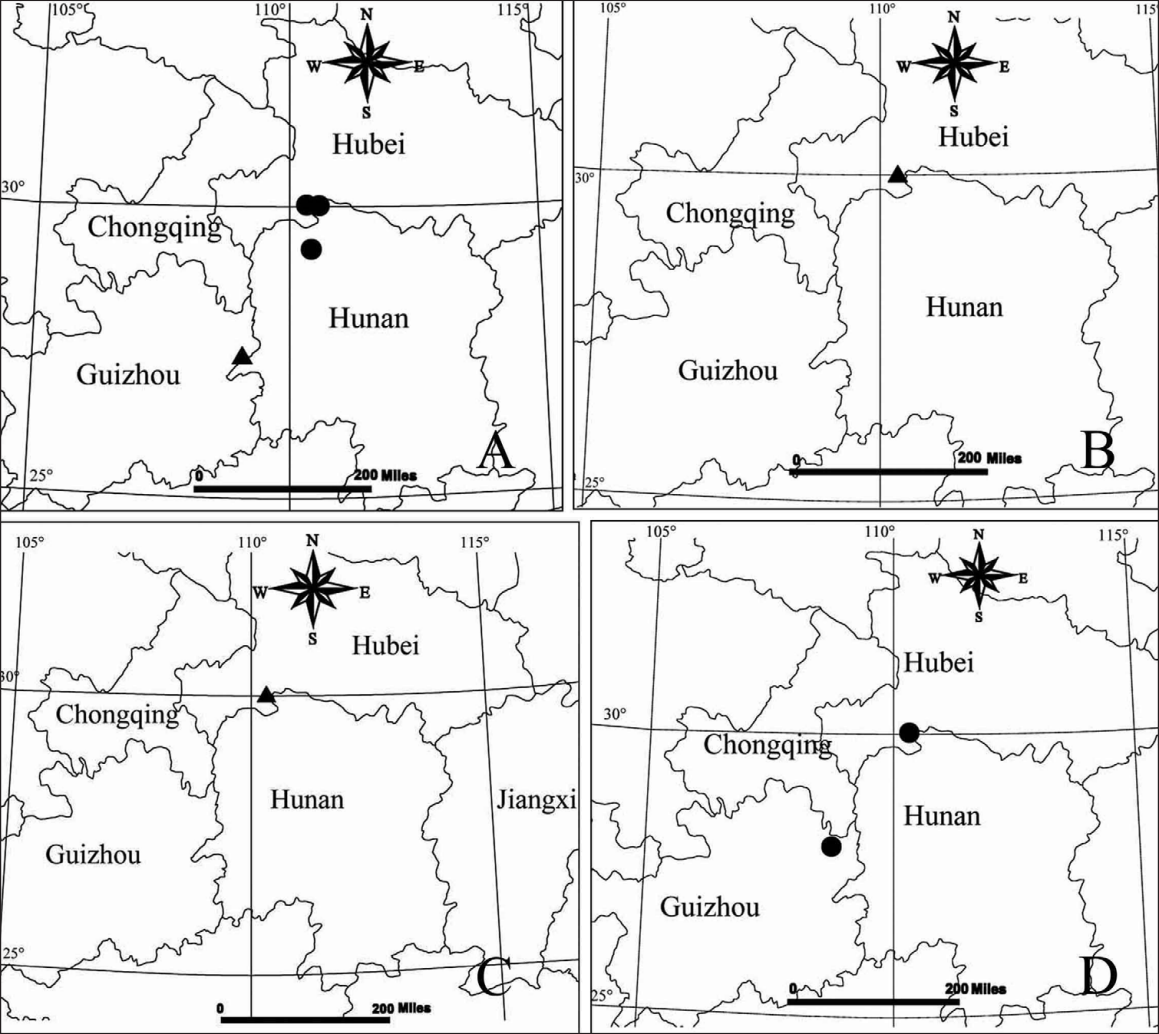
Distribution records of all new species. **A** ● *Phintella arcuata*; ▲ *Phintella pulcherrima* **B** *Phintella levii* **C** *Phintella panda* **D** *Phintella wulingensis*.

## Supplementary Material

XML Treatment for Phintella arcuata

XML Treatment for Phintella levii

XML Treatment for Phintella panda

XML Treatment for Phintella pulcherrima

XML Treatment for Phintella wulingensis

## References

[B1] Barrion AT Litsinger JA (1995) Riceland Spiders of South and Southeast Asia. CAB International, Wallingford, UK, xix + 700 pp.

[B2] Berry JW Beatty JA Prószyński J (1996) Salticidae of the Pacific Islands. I. Distributions of twelve genera, with descriptions of eighteen new species. Journal of Arachnology 24: 214–253.

[B3] Caleb TD J (2014) A new species of *Phintella* Strand (Araneae: Salticidae) from India. Munis Entomology and Zoology 9(2): 605–608.

[B4] Chen CD Li DH (2003) On the biodiversity and the ecological in tegrity of Wulingyuan district, Hunan Prov ince. Acta Ecologica Sinica 23(11): 2415–2423

[B5] Haddad CR Wesolowska W (2013) Additions to the jumping spider fauna of South Africa (Araneae: Salticidae). Genus 24(3-4): 459–501.

[B6] Lei H Peng XJ (2013) Five new species of the genus *Phintella* (Araneae: Salticidae) from China. Oriental Insects 47: 99–110. doi: 10.1080/00305316.2013.783747

[B7] Patoleta B (2009) Description of a new species of *Phintella* Strand in Bösenberg et Strand, 1906 from New Caledonia (Araneae: Salticidae). Genus 20: 539–543.

[B8] Peckham GW Peckham EG (1903) New species of the family Attidae from South Africa, with notes on the distribution of the genera found in the Ethiopian region. Transactions of the Wisconsin Academy of Sciences, Arts and Letters 14: 173–278.

[B9] Peng XJ Xie LP Xiao XQ Yin CM (1993) Salticids in China (Arachnida: Araneae). Hunan Normal University Press, Hunan, China, 270 pp.

[B10] Proszyn’ski J (1979) Systematic studies on East Palearctic Salticidae III. Remarks on Salticidae of the USSR. Annales Zoologici, Warszawa 34: 299–369.

[B11] Prószyński J (1983b) Redescriptions of types of Oriental and Australian Salticidae (Aranea) in the Hungarian Natural History Museum, Budapest. Folia Entomologica Hungarica 44: 283–297.

[B12] Prószyński J (1984a) Atlas rysunków diagnostycznych mniej znanych Salticidae (Araneae). Wyższa Szkola Rolniczo-Pedagogiczna, Siedlcach 2: 1–177.

[B13] Prószyński J (1992) Salticidae (Araneae) of the Old World and Pacific Islands in several US collections. Annales Zoologici, Warszawa 44: 87–163.

[B14] Prószyński J Deeleman-Reinhold CL (2012) Description of some Salticidae (Aranei) from the Malay archipelago. II. Salticidae of Java and Sumatra, with comments on related species. Arthropoda Selecta 21: 29–60.

[B15] Schenkel E (1963) Ostasiatische Spinnen aus dem Museum d’Histoire naturelle de Paris. Mémoires du Muséum National d’Histoire Naturelle de Paris (A, Zool.) 25: 1–481.

[B16] Song DX Gu MB Chen ZF (1988) Three new species of the family Salticidae from Hainan. China. Bulletin of Hangzhou Normal College (nat. Sci.) 1988(6): 70–74.

[B17] Wesolowska W (1981) Salticidae (Aranei) from North Korea. China and Mongolia. Annales Zoologici, Warszawa 36: 45–83.

[B18] Wesolowska W Edwards GB (2012) Jumping spiders (Araneae: Salticidae) of the Calabar area (SE Nigeria). Annales Zoologici, Warszawa 62: 733–772. doi: 10.3161/000345412X659786

[B19] Wesolowska W Russell-Smith A (2000) Jumping spiders from Mkomazi Game Reserve in Tanzania (Araneae, Salticidae). Tropical Zoology 13: 11–127. doi: 10.1080/03946975.2000.10531126

[B20] Wesolowska W Tomasiewicz B (2008) New species and records of Ethiopian jumping spiders (Araneae, Salticidae). Journal of Afrotropical Zoology 4: 3–59.

[B21] Wesolowska W Wiśniewski K (2013) New species of *Phintella* from West Africa (Araneae: Salticidae: Heliophaninae). Genus 24: 247–250.

[B22] Zabka M (1985) Systematic and zoogeographic study on the family Salticidae (Araneae) from Vietnam. Annales Zoologici, Warszawa 39: 197–485.

